# Change process in psychotherapy for criminal offenders: a comprehensive review with content analysis

**DOI:** 10.3389/fpsyg.2026.1776909

**Published:** 2026-05-29

**Authors:** Nihal Tutal, Betul Gokcen Dogan, Gloria Lagetto, Giulio de Felice

**Affiliations:** 1 Department of Psychological Services in Education, Ankara University, Ankara, Türkiye; 2 Department of Educational Sciences, Bozok University, Yozgat, Türkiye; 3 Department of Psychology and Educational Sciences, Pegaso Telematic University, Naples, Italy; 4 Department of Human and Social Sciences, Universitas Mercatorum, Roma, Italy

**Keywords:** change processes, clinical change, criminal offenders, offender rehabilitation, psychotherapy

## Abstract

The aim of this study is to systematically identify and synthesise the therapeutic components that facilitate recovery in criminal offenders undergoing psychotherapy or psychosocial interventions. A systematic review was conducted covering 163 studies published between 2000 and 2024, focusing on interventions such as cognitive-behavioral therapy, schema therapy, mentalisation-based treatment, and attachment-based approaches. A content analysis of these studies resulted in 7 core themes and 32 sub-components representing therapeutic components frequently emphasised in the literature. Structured and directive therapy approaches, cognitive restructuring, and social skills training were the most frequently emphasised, while relational factors such as empathy, warmth, unconditional respect, and therapeutic alliance were less represented. Trauma-focused and cultural or gender-sensitive adaptations were minimal. The findings highlight the predominance of technical, protocol-driven approaches in offender rehabilitation and underscore the need to integrate structured interventions with relational, motivational, and individualised adaptations. Overall, this review provides a comprehensive synthesis of psychological mechanisms discussed in the literature as relevant to offender recovery, emphasising the importance of balanced, multi-component, and individualised therapeutic approaches that address both technical and relational factors.

## 1 Introduction

Criminal offenders are individuals who engage in actions defined as crimes within legal frameworks and who bear criminal responsibility for these actions (Schmalleger, 2023). The behaviors of criminal offenders are not only violations of criminal law but also constitute actions that contradict social norms, ethical principles, and rules that protect social order (Clinard and Meier, 2016). Beyond this normative dimension, however, antisocial behaviour can be understood as a form of relational communication; a nonverbal, often unconscious expression through which the individual signals deep-seated suffering, unresolved trauma, or an unintegrated body–mind structure wrapped-up in fragile psychosocial conditions (Schmideberg, 1947). From this perspective, the offence is not merely a legal or moral transgression but an unconscious attempt to communicate hate, and elicit a response from a social world from which the individual feels profoundly alienated (Orsini, 2023). Recognising such behaviour as a relational act invites a shift from purely punitive responses toward approaches that address the underlying pain and ruptured relationships that frequently lie at its core. Therefore, it is important to evaluate the behaviors of individuals who display criminal conduct comprehensively by considering individual risk factors, family influences, social structures, and developmental processes across the life course (Akers and Sellers, 2021; Andrews and Bonta, 2017).

There are various perspectives that address the offending behaviours of criminal offenders. Psychoanalytic theory has long explored the psychic origins of antisocial behaviour. Klein (1994) work on the genesis of the Super-Ego suggests that early aggressive impulses, persecutory anxieties, and guilt shape later destructive or antisocial actions. Klein argued that some criminal behaviour represents an unconscious attempt to manage overwhelming guilt or repair internal damage through external actions (Klein, 2018).

Winnicott (1968), while influenced by Klein, emphasised disruptions in the environment as a key factor in the development of antisocial tendencies. Winnicott (1968) argued that the antisocial act is paradoxically a “sign of hope”, a plea for the restoration of a previously reliable environment. Psychotherapy with criminal offenders, therefore, requires the recreation of conditions of reliability, containment and emotional holding (Winnicott, 2013).

Schmideberg (1955) expanded on these ideas through her clinical work with offenders, describing the “stable instability” characteristic of many antisocial personalities. In this context, the importance of supporting anticipatory thought was emphasised, as it strengthens individuals’ capacity to evaluate the potential consequences of their actions and to form reliable predictions about environmental responses, thereby enabling the construction of a model of the relationship between the individual and external reality that modulates internal anxiety. This approach aimed to enhance individuals’ tolerance of frustration and interpersonal limits, promoting adaptation to external reality through reflective processes rather than impulsive enactments. Such a relational and flexible approach has anticipated contemporary insights regarding engagement, trust, and the therapeutic alliance in forensic psychotherapy (Schmideberg, 1955).

From a socio-psychological perspective, structural injustice and blocked opportunities also shape violent pathways. Moghaddam’s (2005) “staircase to terrorism” model conceptualises violent action as a narrowing of perceived behavioural alternatives driven by injustice, grievance, and moral disengagement. At the core of such a psychosocial dynamic is the fundamental human need for recognition; specifically, basic narcissistic needs such as being heard, feeling that one’s voice matters, experiencing social integration, and holding a sense of personal value. When the social environment systematically ignores or frustrates these needs, the individual becomes increasingly vulnerable to disengagement from conventional norms and more prone to embracing alternative value systems. These alternatives often emerge within antisocial contexts that valorise dominance, the “law of the strongest”, and a persecutory framing of social relations, offering a distorted but psychologically compelling pathway to agency, significance, and belonging. Although developed in the context of terrorism, the model resonates with clinical theories emphasising how internal meanings interact with external social conditions to produce harmful behaviours.

Taken together, these classical and more contemporary perspectives suggest that criminal act is a dynamic developmental process shaped by early relationships, internal meaning systems, and socio- structural conditions. They also suggest that psychotherapy may offer criminal offenders opportunities for identity reconstruction, affect regulation, relational repair, and meaning-making.

Most criminal offenders eventually return to the community, yet many continue to experience psychological distress, social marginalisation, and elevated risks of reoffending (Bebbington et al. 2021; Cutcher et al., 2014; Thomas et al., 2022). Contemporary justice systems increasingly recognize that risk management alone is insufficient and that the enduring desistance depends on understanding the recovery processes described and experienced by criminal offenders themselves (Maruna, 2001; Nugent and Schinkel, 2016). Parallel to this shift, mental health services have embraced a recovery- oriented paradigm focused on hope, meaning, identity reconstruction, and empowerment (Leamy et al., 2011). However, little is known about the elements of psychotherapy process that facilitate recovery and support desistance (Brine et al., 2021).

Recent empirical studies support the role of psychotherapy in desistance and recovery. Cognitive-behavioural interventions targeting aggression and cognitive distortions reduce reoffending risk (Lipsey et al., 2001). Qualitative studies highlight identity transformation, agency and generative self-narratives as central to sustained desistance (Maruna, 2001; King, 2013). Narrative work shows that criminal offenders who desist often construct “redemption narratives” that allow them to reinterpret past harm and pursue future prosocial goals (Maruna and Roy, 2007).

The recovery paradigm, articulated in the CHIME framework (Connectedness, Hope, Identity, Meaning, Empowerment), has been applied to forensic contexts (Slade et al., 2012; Clarke et al., 2016). Research with forensic psychiatric patients and justice-involved individuals describes recovery as involving trust, relational safety, autonomy, and coping with stigma (Drennan and Wooldridge, 2014; Penney, Morgan, and Simpson, 2021). However, it remains unclear which therapeutic processes are most meaningful.

Despite extensive research on desistance and recovery, to our knowledge, no systematic review has been found that examines the elements of psychotherapy process that influence offender recovery. This study aims to address this gap by synthesising studies examining recovery processes among offenders undergoing psychotherapy or psychosocial treatments (psychotherapy together with social interventions). The objectives of this study are the following:

1. To reveal the therapeutic components that facilitate recovery among criminal offenders (RQ1).
2. To qualitatively synthesise psychotherapy research involving criminal offenders in order to determine the clinical change processes associated with therapeutic improvement (RQ2).

In summary, this study fills a significant gap in the literature by integrating classical developmental perspectives on offenders with contemporary empirical contributions. It clarifies the psychological mechanisms experienced by individuals undergoing psychotherapy during their recovery process, preventing recidivism. Furthermore, by helping to understand the internal transformation of offenders, it offers a powerful social contribution that enhances public safety, reduces stigma, strengthens rehabilitation, and promotes social cohesion.

## 2 Methods

### 2.1 Study design

This study is a comprehensive review of the literature synthesising existing studies focused on clinical change processes of psychotherapy with criminal offenders, preventing recidivism. In contrast with narrative reviews, systematic and comprehensive reviews (Moher et al., 2009), are designed to provide an integrated and objective overview of the available evidence, providing a final synthesis of the results. This approach reinforces the reliability of research findings and is particularly salient in evidence-based psychological research and clinical practice (Page et al., 2021).

### 2.2 Search strategies

The search strategy was iteratively refined to balance sensitivity and specificity and to ensure adequate representation of different therapeutic modalities used with offender populations. Electronic searches were conducted across multiple databases, including comprehensive searches of (1) PsycINFO (APA), (2) Web of Science Core Collection, (3) Scopus, (4) PubMed/MEDLINE, and (5) Criminal Justice Abstracts. Search terms were constructed using combinations of keywords related to criminal offending and psychotherapy, including “criminal offenders,” “offender psychotherapy,” “forensic psychotherapy,” “psychological treatment of offenders”, “clinical change processes”, “therapeutic alliance”, “risk-need-responsivity”, “schema therapy”, “mentalisation-based treatment”, and “dialectical behavior therapy”. Boolean operators (AND/OR) were used to combine search terms. The search was limited to publications from January 1, 2000 to December 31, 2024. To enhance methodological transparency and reproducibility the full search strings, coding procedures, and article coding tables have been provided as Supplementary material.

### 2.3 Inclusion and exclusion criteria

This work includes research articles that were published between 2000 and 2024, with literature that had not been published in English left out. The search included systematic reviews, RCTs, and theoretical studies of the foundational models (e.g., key articles about the Risk-Need-Responsivity Model of Corrections or the Good Lives Model of therapy), alongside empirical studies investigating clinical change processes. Candidate articles must be related to the effectiveness and clinical processes of psychological approaches (e.g., cognitive behavior therapy, neuropsychological therapies, motivational approaches, psychodynamic therapies, or attachment- based approaches) implemented with convicted individuals (e.g., inmates, sexual offenders, offenders with mental health related problems). The type of offender populations that were investigated in the study included those that had personality disorders (PD), sexual offenders, and individuals who had dual diagnoses of antisocial personality disorder (ASPD) and substance use. The core inclusion criteria of this study was that the empirical or clinical evidence of the clinical change process must be evident in the publication. Contributions focused on offenders with intellectual disability and on offenders who had committed property crimes were excluded. Clearly, research that did not investigate the clinical change processes were also left out.

### 2.4 Study selection process

**Figure 1:**
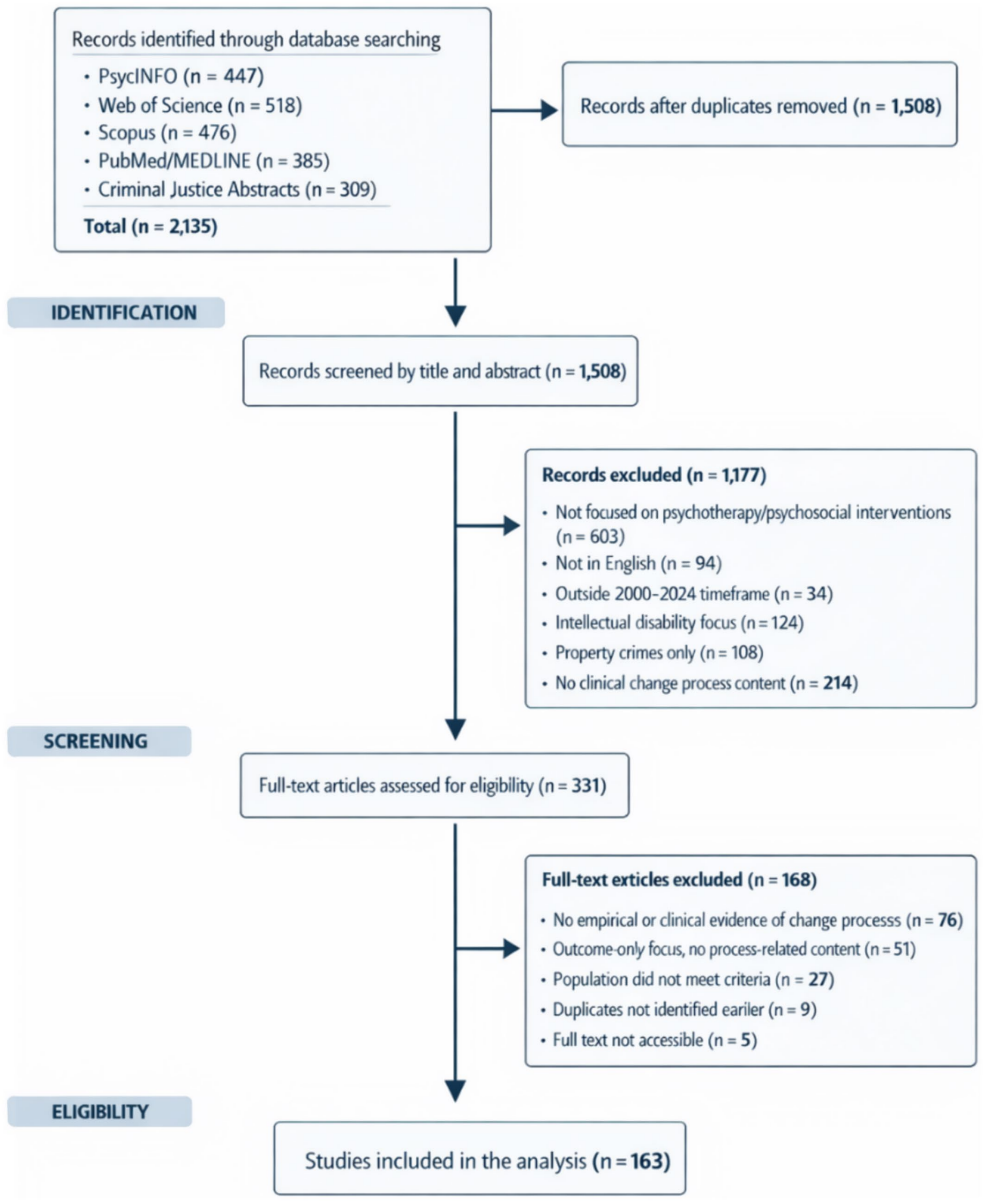
PRISMA flow diagram of the study selection process.

The study selection process followed the PRISMA 2020 guidelines (Page et al., 2021). Initial database searches across PsycINFO, Web of Science Core Collection, Scopus, PubMed/MEDLINE, and Criminal Justice Abstracts yielded 1,508 records after duplicates were removed. Title and abstract screening was conducted independently by two authors, with disagreements resolved through discussion. Following this, 331 full-text articles were assessed for eligibility independently by the same authors. After applying the inclusion and exclusion criteria, a total of 163 studies were included in the final content analysis. A PRISMA flow diagram summarising the selection process is presented in Figure 1.

### 2.5 Data analysis

No review protocol was registered for this systematic review (e.g., with PROSPERO). The coding framework was developed inductively after the study selection process was completed. In this study, a content analysis approach was used to identify the active therapeutic components that facilitate change in criminal offenders, based on the examination of 163 studies. An initial coding framework was inductively developed, comprising 7 themes and 32 subthemes, based on established theoretical concepts present throughout the literature such as the Risk-Need-Responsivity (RNR) model, Cognitive Behavioral Therapy (CBT), and the Good Lives Model (GLM). Specific English keywords were identified for each subtheme. The search results from each study were systematically reviewed row-by-row in Excel and coded for the presence of these keywords, allowing a single study to contribute to multiple components.

To ensure coding reliability, a randomly selected subset of 30 studies (18.4% of the total sample) was independently coded by two researchers. Cohen’s Kappa was calculated separately for each of the 32 subcomponents. The mean Kappa across all subcomponents was 0.92, with values ranging from 0.79 to 0.97. The highest agreement was observed for concrete subcomponents such as I.4 Structured and Directive Therapy (*κ* = 0.97) and IV.1 Social Skills Training (κ = 0.95). Lower but still acceptable agreement was observed for more abstract subcomponents such as II.3 Building Hope and Optimism (κ = 0.81) and VI.2 Therapist’s Personal Values and Attitudes (κ = 0.79). All coding was based on full-text reading and contextual verification (as detailed in Supplementary material C). Coding and reliability calculations were performed using Microsoft Excel with manual verification. Following coding, the frequency of each component, defined as the number of studies in which it appeared, was calculated and converted into a percentage of the total sample (*N* = 163). Components were then ranked by frequency. All included studies, regardless of design (empirical or theoretical), were coded using the same framework, as our aim was to map the emphasis placed on different therapeutic components across the literature rather than to weight findings by study design.

To enhance analytical rigor, keyword searches were repeated, and any coding discrepancies were resolved through discussion until consensus was reached. This structured yet iterative approach enabled a systematic quantification of the emphasis placed on different treatment components within the literature.

## 3 Results

This section presents the findings of the content analysis conducted on 163 studies examining therapeutic change processes in psychotherapy for criminal offenders. First, the characteristics of the included studies are summarised to provide context for the subsequent findings. A detailed overview of study design, population type, intervention modality, setting, and focus is presented in Table 1.

**Table 1:** Characteristics of included studies (*N* = 163).

Characteristic	Category	*n*	%
Study design	Review article/theoretical	98	60.1
	Systematic review/meta-analysis	31	19.0
	Observational/longitudinal	13	8.0
	Qualitative	12	7.4
	Randomized controlled trial (RCT)	7	4.3
	Quasi-experimental	2	1.2
Population type	Sexual offenders	51	31.3
	Mixed/general offenders	47	28.8
	Psychopathy/ASPD	23	14.1
	Mentally disordered offenders	11	6.7
	Personality disorder	9	5.5
	Juvenile offenders	8	4.9
	Violent offenders	7	4.3
	Substance use/co-occurring	5	3.1
	Domestic violence	2	1.2
Intervention modality	Multi-modal/other	77	47.2
	Cognitive behavioral therapy (CBT)	34	20.9
	Good lives model (GLM)/strengths-based	12	7.4
	Schema therapy	10	6.1
	Psychodynamic/psychoanalytic	8	4.9
	Mentalisation-based therapy (MBT)	5	3.1
	Risk-need-responsivity (RNR)	5	3.1
	Therapeutic community	5	3.1
	Dialectical behavior therapy (DBT)	4	2.5
	Arts therapy	2	1.2
	Motivational interviewing	1	0.6
Setting	Not specified/review	47	28.8
	Mixed/multiple settings	44	27.0
	Prison	42	25.8
	Forensic hospital/inpatient	15	9.2
	Community	15	9.2
Focus	Outcome-focused	92	56.4
	Process-focused	26	16.0
	Mixed	23	14.1
	Theoretical	22	13.5

Study characteristics were coded based on information extracted from each included article. Percentages may not sum to 100 due to rounding.

As shown in Table 1, the majority of studies were review articles or theoretical contributions (60.1%), followed by systematic reviews and meta-analyses (19.0%). Observational and longitudinal studies accounted for 8.0%, qualitative studies for 7.4%, randomized controlled trials for 4.3%, and quasi-experimental studies for 1.2%.

In terms of population, sexual offenders were the most frequently examined group (31.3%), followed by mixed or general offender samples (28.8%), individuals with psychopathy or antisocial personality disorder (14.1%), mentally disordered offenders (general definition, no specific diagnosis) (6.7%), and those with personality disorders (5.5%). Juvenile offenders (4.9%), violent offenders (4.3%), individuals with substance use or co-occurring disorders (3.1%), and domestic violence offenders (1.2%) were also represented.

Regarding intervention modality, multi-modal or unspecified approaches were most common (47.2%), followed by cognitive-behavioral therapy (20.9%). Good Lives Model or strengths-based approaches (7.4%), schema therapy (6.1%), psychodynamic or psychoanalytic approaches (4.9%), mentalisation-based treatment (3.1%), risk-need-responsivity-based interventions (3.1%), and therapeutic communities (3.1%) were also represented. Dialectical behaviour therapy (2.5%), arts therapies (1.2%), and motivational interviewing (0.6%) appeared less frequently.

The largest proportion of studies did not specify the therapeutic setting (28.8%), while mixed or multiple settings accounted for 27.0%. Prison-based studies represented 25.8%, while forensic hospital or inpatient settings and community-based settings each accounted for 9.2%.

Outcome-focused studies (primarily examining recidivism) were predominant (56.4%), followed by process-focused studies examining therapeutic mechanisms (16.0%). Process-outcome studies accounted for 14.1%, while purely theoretical contributions accounted for 13.5%.

The content analysis of 163 articles extracted from the literature review gave rise to 32 subcomponents; from them 7 core themes commonly discussed as relevant to change in psychotherapy were abstracted. The core themes and their subcomponents are as follows:

Theme I: Components Related to *Therapeutic Relationship and Alliance (5 subcomponents)* This theme highlights relational factors such as empathy, trust, collaboration, structure, and supportive elements of psychotherapy process (Marshall et al., 2003; Kozar and Day, 2012; DeSorcy et al., 2017).
Theme II: Components Related to *Motivation and Readiness to Change (5 subcomponents)* This theme includes stage-based motivational interventions, motivational interviewing, hope-building strategies and motivational balancing (Martin and Stermac, 2010; Moulden and Marshall, 2005).
Theme III: *Cognitive and Emotional Regulation Components (5 subcomponents)* - This theme includes cognitive restructuring, schema processing, emotion regulation, mindfulness and mentalisation (Bernstein et al., 2007; Chakhssi et al., 2013; Yates and Ward, 2008).
Theme IV: *Behavioural and Social Skill Components (5 subcomponents)* - This theme focuses on behavioural and social skill components such as social skills training, problem solving, anger management, relapse prevention, and social support (McCann et al., 2000; Wetterborg et al., 2018).
Theme V: *Group and Environmental Support Components (4 subcomponents)* - This theme stresses the value of group and environmental support, i.e., it includes group binding, community and family encouragement; safe institutional climate, trust in institutions (DeCarlo and Hockman, 2004; Hobson et al., 2000).
Theme VI: *Therapist Characteristics and Quality of Psychotherapy Implementation (4 subcomponents)* - The practitioner-level category includes therapist’s elements, i.e., therapist’s competence, values, therapeutic trustworthiness and supervision (Bach and Demuth, 2019; Drapeau, 2005).
Theme VII: *Individualised Adaptation (Responsivity) (4 subcomponents)* - This theme concerns risk responsivity, cultural/gender responsivity, responsivity and adaptation to severe personality disorders and traumatic experiences (Howells and Day, 2006; Looman et al., 2005).

Based on this thematic framework, the number of keywords of each study was analysed in terms of frequency of components and subcomponents weighted across the total of 163 papers (Table 2).

**Table 2:** Ranking and frequency of components and subcomponents of the change processes.

Rank	Theme	Subcomponents	Frequency (%)	Rank	Theme	Subcomponents	Frequency (%)
1	I.4	Structured and Directive Therapy (SDT)	52 (%31.90)	17	VII.3	Adaptations for Psychopathy and Personality Disorders	18 (%11.04)
2	III.1	Identifying and Modifying Cognitive Distortions (IMCD)	42 (%25.77)	18	III.5	Enhancing Mentalisation Capacity	16 (%9.82)
3	IV.1	Social Skills Training (SST)	38 (%23.31)	19	IV.5	Social Integration Skills	16 (%9.82)
4	III.3	Developing Emotion Regulation Skills (DERS)	35 (%21.47)	20	II.1	Interventions Tailored to Stages of Change	15 (%9.20)
5	I.1	Empathy, Warmth, and Respect (EWR)	29 (%17.79)	21	VI.2	Therapist’s Personal Values and Attitudes	17 (%10.43)
6	IV.2	Problem Solving and Decision Making Skills (PSDMS)	27 (%16.56)	22	I.2	Trust and Safety Feeling	14 (%8.59)
7	IV.4	Developing Relapse Prevention Plans (DRPP)	27 (%16.56)	23	III.4	Mindfulness and Acceptance Techniques	14 (%8.59)
8	VII.1	Intensity Adjustment Based on Risk Level (IABRL)	25 (%15.34)	24	II.5	Combining External Incentives with Internal Motivation	13 (%7.98)
9	VI.1	Therapist Competence and Training (TCT)	24 (%14.72)	25	II.3	Building Hope and Optimism	12 (%7.36)
10	I.3	Collaboration and Setting Shared Goals	23 (%14.11)	26	V.3	Family and Social Environment Involvement	12 (%7.36)
11	II.2	Motivational Interviewing Techniques	22 (%13.50)	27	III.2	Processing Early Maladaptive Schemas	11 (%6.75)
12	IV.3	Anger Management and Aggression Control	21 (%12.88)	28	V.4	Institutional Climate and Safety Feeling	10 (%6.13)
13	II.4	Setting Positive Goals with the GLM	19 (%11.66)	29	VI.4	Supervision and Support Systems	10 (%6.13)
14	VI.3	Therapeutic Fidelity and Manual Adherence	19 (%11.66)	30	V.2	Community-Based Support Networks	9 (%5.52)
15	I.5	Supportive Challenging Rather than Confrontation	18 (%11.04)	31	VII.2	Sensitivity to Cultural, Gender-Specific Needs	9 (%5.52)
16	V.1	Group Cohesion and Solidarity	18 (%11.04)	32	VII.4	Working with Trauma History	7 (%4.29)

Operational definitions for each subcomponent are provided in Supplementary material C. Each theme is numbered with Roman numbers; subcomponents are numbered with Arabic numbers. Sub-components are listed in order of their frequency of occurrence.

The frequency data presented in Table 2 reveal the relative emphasis placed on the different therapeutic change components. The most frequently cited component was Structured and Directive Therapy (SDT) (I.4), identified in 31.90% (*n* = 52) of the reviewed studies. This finding highlights the central importance accorded to treatment structure, manual adherence, and intervention fidelity within offender rehabilitation frameworks (Pearson et al., 2002).

On the other hand, the heterogeneity of interventions discussed in the literature is reflected in the other highly ranked components. Identifying and Modifying Cognitive Distortions (IMCD; 25.77%) (III.1) and Social Skills Training (SST; 23.31%) (IV.1) ranked second and third, respectively, underscoring the prominence of cognitive-behavioural techniques. These were followed closely by Developing Emotion Regulation Skills (DERS; 21.47%) (III.3) and the relational component of Empathy, Warmth, and Respect (EWR; 17.79%) (I.1). The co-occurrence of these technical and relational elements among the top five indicates a multidimensional understanding of change.

Other components, such as Problem-Solving and Decision-Making Skills (PSDMS) (IV.2), Developing Relapse Prevention Plans (DRPP) (IV.4), Intensity Adjustment Based on Risk Level (IABRL) (VII.1), and Therapist Competence and Training (TCT) (VI.1), appeared in approximately 14–16% of studies, suggesting that effective rehabilitation also hinges on skill application, individualised risk management, and practitioner expertise.

Content analysis further clarifies these patterns. While Themes I (Therapeutic Relationship and Alliance; total: 21.18%; components in the top 10 ranks: 31.90, 17.79, and 14.11%), III (Cognitive and Emotional Regulation; total: 18.38%; components in the top 10 ranks: 25.77 and 21.47%), and IV (Behavioural and Social Skills; total: 20.09%; components in the top 10 ranks: 23.31, 16.56, and 16.56%) contain the highest concentration of frequently mentioned components, Theme II (Motivation and Readiness to Change; total: 12.62%; highest-ranked component: 13.50%, appearing only at rank 11) and its subcomponents never occurred once within the first 10 ranks. This may reflect the stronger emphasis on treatment guidelines and adherence, and that difficulties remain in identifying effective ways to engage and motivate this clinical population.

Furthermore, to assess whether the inclusion of theoretical studies influenced the frequency rankings, a sensitivity analysis was conducted excluding purely theoretical papers (*n* = 98). When the analysis was restricted to empirical studies only (*n* = 65), the ranking of the top five components remained largely unchanged: Structured and Directive Therapy (36.9%), Identifying and Modifying Cognitive Distortions (35.4%), Social Skills Training (23.1%), Developing Emotion Regulation Skills (21.5%), and Empathy, Warmth, and Respect (20.0%). This suggests that the overall pattern of emphasis is robust and not unduly influenced by the inclusion of theoretical contributions. Notably, Therapist Competence and Training (36.9%) and Therapeutic Fidelity and Manual Adherence (29.2%) emerged with greater prominence in the empirical subset,. This indicates that issues such as therapist competence and therapeutic collaboration, which focus on the practitioner and the implementation, are addressed more clearly and explicitly in empirical studies.

**Figure 2:**
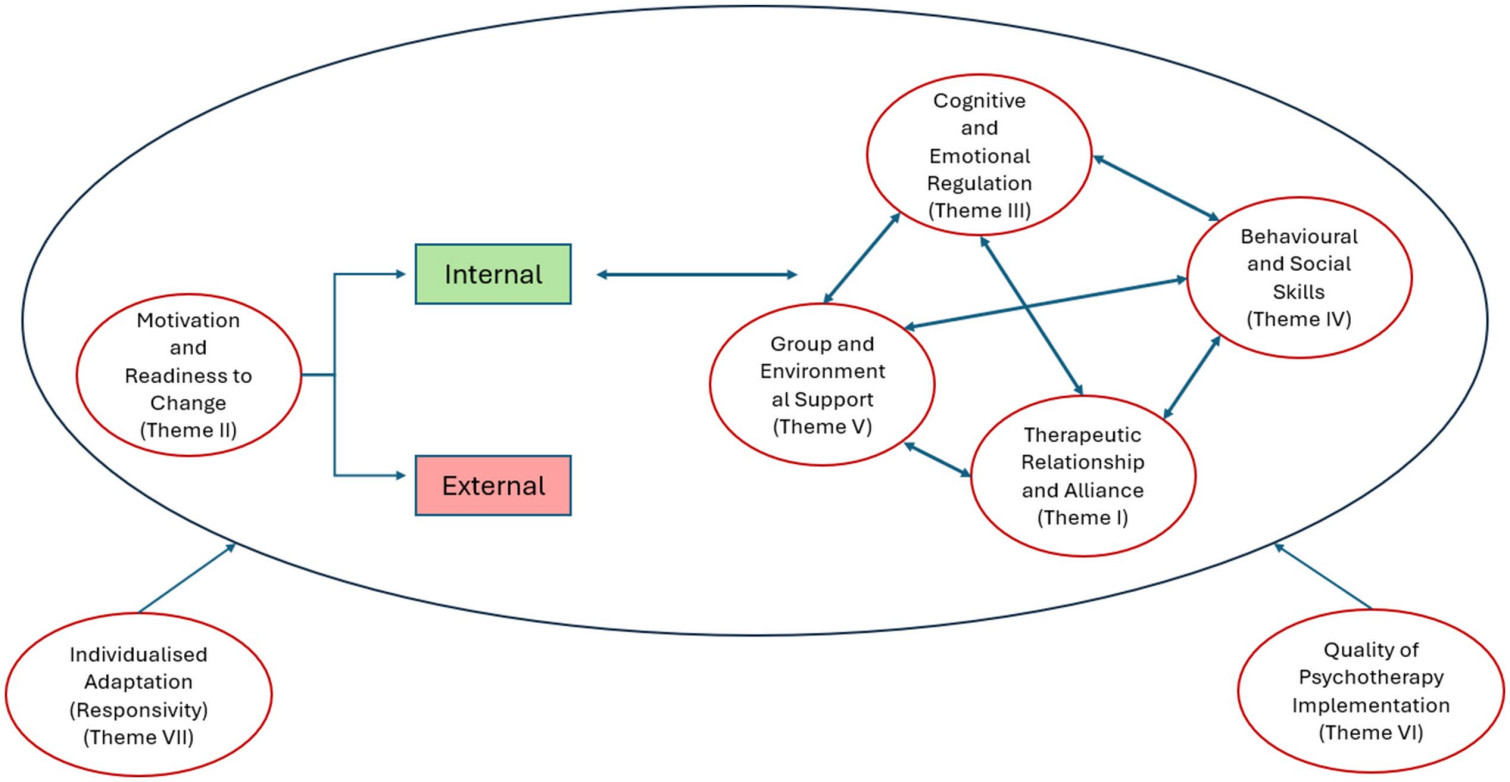
Schematic representation of the connection among the 7 themes and the therapeutic change model that they entail. Motivation and readiness to change frame the therapeutic intervention in terms of external versus internal motivation. The latter constitutes the main foundation upon which therapeutic change can occur. The network of themes I, III, IV, V is composed of “change-inducing mechanisms” and “reinforcing factors.” The good functioning of such a network consolidates internal motivation, and vice versa. Finally, the quality of psychotherapy implementation and the individualised adaptation ensure the good functioning of the entire system.

Collectively, the results suggest that therapeutic change in offenders is not attributable to isolated components. Instead, the literature supports an integrated, multimodal model of change that combines structured skill-building, a strong therapeutic relationship promoting self-regulation, motivational support and risk-aware individualisation. A schematic representation of the connection among the 7 themes and the general therapeutic change model that they entail is presented in Figure 2.

Motivation and readiness to change frame the therapeutic intervention in terms of external versus internal motivation. The latter (indicated in green) constitutes the main foundation upon which therapeutic change can occur. External and internal motivation should not be understood as a dichotomy but rather as a continuous variable, constantly influenced by the therapeutic intervention (represented by the thematic network on the right side of Figure 2). The more this intervention enables the working through of the patient’s core internal anxiety, the more internal motivation is consolidated and the patient’s trust in the therapeutic process increases. The network of themes I, III, IV, V is composed of “change-inducing mechanisms” and “reinforcing factors.” The “change-inducing mechanisms” are:

(a) *Cognitive Restructuring*. It represents a crucial component of therapeutic change in antisocial individuals, as it directly targets the distorted beliefs, justifications, and cognitive biases that sustain offending behaviour. However, its application is often far from being straightforward. For patients with severe antisocial trajectories, particularly those who have experienced detention, cognitive interventions encounter a deeply entrenched value system that is not merely a collection of distortions but an alternative, internally coherent moral framework. This framework frequently prioritises loyalty to in-group codes, the logic of retaliation, and a rejection of conventional social norms, effectively rendering prosocial values alien or threatening. In such cases, cognitive restructuring cannot simply aim to replace “irrational” thoughts with adaptive ones; it must first engage with a value system that has provided structure, identity, and survival utility within criminal offenders and sub-group contexts. The therapeutic challenge intensifies when antisocial features co-occur with psychosis. In these patients, cognitive rigidity may be compounded by delusional elaboration, impaired reality testing, and a fragmented capacity for therapeutic alliance, making the collaborative examination of beliefs and values particularly demanding. Thus, while cognitive restructuring is indispensable, its effectiveness in this population hinges on a gradual, relationally grounded process that acknowledges the adaptive roots of antisocial cognition and the additional layers of complexity introduced by severe mental disorders.

(b) *Emotion Regulation*. It constitutes another fundamental target of therapeutic work with antisocial patients, yet it is difficult to achieve. In this population, emotional functioning often operates in a discrete, dichotomous manner: patients oscillate between profound emotional anesthesia, a numbing that shields them from vulnerability, and sudden, overwhelming outbursts of hate, rage or uncontrolled violence. This polarised pattern reflects deep deficits in the capacity to identify, tolerate, and modulate affective states, rendering interpersonal relationships volatile and trust precarious. In such cases, the secure environment of detention can serve not merely as a custodial measure but as a necessary therapeutic container. The physical and relational safety provided by a structured setting allows the patient’s persecutory anxiety to gradually subside, creating the foundational conditions in which therapeutic work can begin. Effective containment, offered consistently by staff and therapists, helps to de-escalate hypervigilance and paranoid projections, thereby making it possible to establish a relationship based on mutual trust. Only within such a holding environment can emotion regulation skills be meaningfully introduced, practiced, and integrated, moving the patient beyond the stark extremes of affective shutdown or explosive dyscontrol.

(c) *Identification with Social Values*. It represents a pivotal dimension of therapeutic change in criminal offenders. As the sociological literature on radicalisation processes consistently demonstrates (Orsini, 2023), such individuals identify with a coherent system of “anti-values” those of the antisocial sub-group, including codes of silence (omertà), the legitimisation of violence, and a pervasive persecutory worldview. Shifting this identification toward prosocial values, therefore, requires a profound transformation in the objects of loyalty and belonging. When a patient begins to identify with values such as reciprocity, mutual responsibility, and respect for the law, they gain the capacity to experience themselves as socially integrated, a fundamental narcissistic need that the antisocial identity had previously fulfilled with the identification with the antisocial sub-group. Achieving this shift is greatly facilitated by synergy with concrete social reintegration interventions, particularly those focused on employment and stable social roles. Such interventions provide tangible external objects, colleagues and institutions, with which the individual can form new, prosocial identifications. Ideally, a well-coordinated psychosocial intervention offers the patient the opportunity to restructure the constellation of external/internal objects they identify with, gradually replacing the antisocial reference group with a network of relationships and institutions that foster a sustainable sense of belonging.

Finally, the *reinforcing factors* play an essential role in consolidating the prosocial identity of former criminal offenders, ensuring that therapeutic gains are sustained beyond the treatment setting. Among the most influential of these factors are the quality of prosocial bonds within the offender’s community and the degree of genuine social integration they experience. When individuals are embedded in networks that offer stable employment, constructive social roles, and relationships grounded in mutual accountability, the new prosocial identity is continuously affirmed and strengthened. Equally critical are external validation messages, explicit or implicit communications from family members, colleagues, therapists, or community members that convey the “you have changed” messages. Such recognitions serve as powerful social mirrors, helping the individual internalise their transformation and feel that their new identity is seen, acknowledged, and appreciated by others. In the absence of these reinforcing elements, the risk of relapse into antisocial patterns and identifications increases markedly. Thus, a comprehensive intervention must attend not only to intrapsychic change but also to the environmental and relational conditions that sustain and reinforce the prosocial identity over time.

## 4 Discussion

This study sought to identify the therapeutic components facilitating recovery in criminal offenders (RQ1) and to identify the clinical change processes associated with therapeutic improvement (RQ2). A comprehensive review with content analysis of 163 studies yielded a framework of 7 core themes and 32 sub-components. It is important to note that the frequency of particular themes does not necessarily correspond to the robustness or strength of empirical evidence, but may reflect research trends. The quantitative findings reveal a clear hierarchy of emphasis within the literature: *structured and directive therapy* (31.90%), *cognitive restructuring* (25.77%), and *social skills training* (23.31%) are the most prominent. This aligns with established evidence suggesting that cognitive-behavioral and structured programs are associated with lower recidivism rates (Pearson et al., 2002; Smith et al., 2024). The high frequency of these components may reflect the field’s strong orientation toward protocol-driven, skill-based intervention (Syasyila et al., 2024). The therapeutic setting seems to play a central role for this clinical population, given that structured and clearly defined environments support individuals who commonly experience difficulties related to interpersonal consistency, test therapeutic boundaries, and instability within developmental and social context. A consistent therapeutic frame may foster a sense of safety and containment, while also supporting emotional regulation and continuity with the therapeutic process. From this perspective, the therapeutic setting may be understood as a factor associated with therapeutic change rather than merely a neutral background.

On the other hand, the ranking of social skills training (SST) at 23.31% reflects the nuanced and sometimes inconsistent evidence regarding its impact. While programs such as EQUIP (Equipping Youth to Help One Another) or Aggression Replacement Training have been associated with behavioural and psychosocial improvements, findings regarding their relationship with recidivism remain mixed. This variability may depend on factors such as research design, participant age - with some studies reporting stronger associations among adolescents - and whether SST is delivered as a standalone intervention or as part of a broader, multi-component treatment protocol (der Van Stouwe et al., 2021). For instance, skill-building programs for juvenile offenders can show significant reductions in reoffending under specific conditions, whereas outcomes for adult populations are often more modest and inconsistent. Consequently, the present finding is consistent with the view that SST, while beneficial, may have limited sustained impact when implemented in isolation. This underscores a key principle in the literature: the most effective interventions are generally multi-modal, integrating structured cognitive-behavioral components and social skills training into a unified protocol (Pappas and Dent, 2023). Thus, the high frequency of social skills training, cognitive restructuring, and structured therapy identified in this review should not be interpreted in isolation; instead, it may suggest the importance of integrating these complementary elements within a broader psychosocial intervention.

The secondary ranking of *relational factors*—such as empathy, warmth, unconditional respect, and the therapeutic alliance (17.79%)—presents a critical point for reflection. While meta-analyses confirm these elements are vital for treatment engagement and reducing recidivism (Hoeve et al., 2012; Karver et al., 2018), their lower prominence in the literature suggests a potential undervaluation of the relational matrix in which technical change occurs. This interpretation is confirmed by the discrepancy in the frequencies of the sub-themes of Theme 1. within this theme, there is a notable imbalance: the structural component I.4 Structured and Directive Therapy appears in 31.90% of studies, whereas the affective-relational components—I.1 Empathy, Warmth, and Respect (17.79%), I.2 Trust and Safety Feeling (8.59%), I.3 Collaboration and Setting Shared Goals (14.11%), and I.5 Supportive Challenging Rather than Confrontation (11.04%)—together account for a smaller proportion. From a psychoanalytic perspective, this discrepancy is clinically significant. The therapeutic alliance provides the essential holding environment (Winnicott, 1968) necessary for containment. For individuals with antisocial tendencies, whose actions may paradoxically signal a “hope” for environmental reliability (Winnicott, 2013), the consistent, empathic presence of the therapist is not merely supportive but structurally reparative. It allows for the internalisation of a stable object, mitigating the “stable instability” described by Schmideberg (1955). Furthermore, cognitive restructuring of distortions can be reframed psychoanalytically as the delicate work of addressing persecutory anxieties and modifying harsh, primitive superego functions, akin to Klein (1994) explorations of early aggressive impulses and guilt.

The relatively low emphasis of *motivational components* (e.g., Motivational Interviewing at 13.50%) and strengths-based models like the Good Lives Model (11.66%) indicates the enduring dominance of a deficit-focused, risk-management paradigm. However, motivational interviewing, has presented evidence in recent years as an effective method for increasing treatment participation in samples of criminal offenders with low motivation and resistance levels (Van Damme et al., 2015; Miller and Rollnick, 2012). On the other hand, the Good Lives Model, focusing on individuals’ strengths, basic psychological needs, and well-being offers a significant complementary framework to the classic RNR (Risk-Need-Response) model, which focuses solely on risk reduction (Ward and Maruna, 2007). Indeed, recent research shows that interventions integrating GLM and RNR increase both motivation and adherence to treatment; they not only reduce problematic behaviours but also contribute to individuals building a more sustainable, pro-social life (Ward and Fortune, 2016; Willis et al., 2013). However, the emergence of treatment adaptations for personality disorders and psychopathy (their prevalence is 11.04%), including schema therapy and mentalisation-based treatment, marks a crucial advancement in the clinical practice (Bernstein et al., 2012; Evershed et al., 2003; McGauley et al., 2011); in fact, these approaches successfully integrate psychodynamic principles—such as processing early maladaptive schemas (III.2) and enhancing reflective capacity demonstrating that the clinical work on affects, promoting the revision of relational schemata, can effectively engage the depth-psychological structures underlying chronic offending.

The lowest-ranked components—*trauma-focused interventions* (4.29%) and *cultural/gender specific adaptations* (5.52%)—highlight the most substantial gaps in both research and, by extension, clinical practice. The limited representation in the literature of specific interventions developed for female offenders and minority groups may suggest a lack of social justice in the general approach to rehabilitation (Bloom et al., 2003; Covington, 2008; Lewis-Fernández et al., 2017). This minimal representation suggests that the etiological roots of much offending behaviour, often embedded in unresolved trauma and social marginalisation, are insufficiently addressed. Psychoanalytically, the antisocial act can be understood as a form of *enactment,* a somatic discharge of unprocessed traumatic experience, with the aim of testing the environment in terms of its capacity to listen to the patient’s ego needs and its capacity to firmly keeping the relational limits while avoiding violence (process of individualisation) (de Felice et al., 2020; de Felice & Tutal, 2023). Without therapeutic spaces dedicated to processing these experiences, interventions on risk reduction remain superficial, failing to address the internal conflicts and split-off self-states that drive behaviour. Similarly, the lack of focus on *social reintegration components*, such as community-based support networks (5.52%), reflects an individualistic model that neglects the vital role of the social environment in sustaining recovery, overlooking the need for a supportive “community of care” to facilitate genuine desistance.

In direct response to RQ1, this review suggests that recovery is supported by a multifaceted model in which structured, cognitive-behavioural components and social skills training are most prominently represented. In response to RQ2, the findings frame the clinical change process as a complex and multi-layered phenomenon (Figure 2). Within this framework, three interrelated “change-inducing mechanisms” were frequently identified: (a) cognitive restructuring, which addresses the distorted beliefs and justifications sustaining antisocial conduct; (b) emotion regulation, which enables the individual to move beyond the extremes of affective anesthesia and explosive dyscontrol; and (c) identification with social values, which involves a profound reorientation away from the anti-values of the antisocial sub-group toward a sense of belonging grounded in prosocial norms. These mechanisms appear unlikely to operate independently. Rather, their maintenance may also be associated with reinforcing contextual factors that support the development of a prosocial identity. Among these factors, stable prosocial relationships within the community and experiences of meaningful social integration were frequently highlighted, particularly in relation to the alienation often described in antisocial trajectories. Taken together, these findings suggest that recovery in this population may involve an ongoing interaction between therapeutic processes and the broader environmental and relational conditions that support prosocial development over time.

Finally, the findings support the value of a more integrated framework that balances the technical fidelity with the attention to affective and relational dimensions of clinical work, with a specific focus on persecutory anxieties. Within such a clinical frame, the experiences of being understood within reliable therapeutic boundaries contribute to meaningful psychological change among forensic patients (Figure 2).

### 4.1 Suggestions for future research

Future studies could employ qualitative and mixed-method designs to conduct in-depth investigations of therapeutic change processes. In order to understand the mechanisms of change it is relevant to investigate the clinical and empirical precursors of change. In psychotherapy research, the stability-flexibility oscillations (S-F Oscillations) among the process variables revealed to be particularly significant in predicting change (de Felice, 2024; de Felice et al., 2025). Of course this perspective requires longitudinal studies as an essential framework to track desistance processes and to investigate which intervention components lead to lasting behavioural change and successful social reintegration. On this topic future research should investigate the processes through which identification with positive, community-oriented values can be nurtured. From a psychoanalytic standpoint, this is not merely a matter of behavioural training but a profound endeavour of identity reworking. Antisocial values are frequently crystallised as Ego defenses against early trauma, humiliation, or environmental failure. Fostering a stable identification with new, prosocial values, therefore, requires not only cognitive learning but a slow and complex process of introjecting new internal objects, made possible only within a secure and enduring therapeutic affective encounter. This is particularly challenging in adult populations, where personality structures and defensive patterns are more entrenched.

Finally, the effectiveness of underrepresented components, such as trauma-focused interventions, cultural and gender-sensitive adaptations, must be empirically investigated with outcome measures that extend beyond symptomatology, to clarify subgroup-specific needs and differential conditions for clinical interventions.

### 4.2 Limitations

These findings should be interpreted in light of some limitations. First, the review was based on a qualitative synthesis of the literature, and the findings are therefore tied to the specific body of studies analysed rather than being directly generalisable to other clinical populations. In addition to empirical studies, the review also included theoretical, conceptual, and secondary literature. While the inclusion of these sources enabled a broader and more integrative understanding of therapeutic processes and recovery mechanisms among criminal offenders, it may also have introduced interpretative heterogeneity. In particular, some of the explanatory mechanisms discussed in the review are theoretically informed and the empirical evidences supporting them may vary. Furthermore, criminal offenders constitute a heterogeneous population, and the findings presented here reflect aggregate patterns across diverse subgroups. Future research may benefit from examining whether the relative emphasis placed on specific therapeutic components differs across diagnostic and offender subtypes. The appendix provided in the Supplementary material includes the full list of the 163 articles included in the review.

## Data Availability

The datasets presented in this study can be found in online repositories. The names of the repository/repositories and accession number(s) can be found in the article/Supplementary material.
