# Supplementary material for: Change process in psychotherapy for criminal offenders: a comprehensive review with content analysis
Source: Front Psychol. 2026 May 29;17:1776909. doi: 10.3389/fpsyg.2026.1776909 (PMC13260342; doi:10.3389/fpsyg.2026.1776909)
Supplement: Supplementary file 2 [file Supplementary_file_2.DOCX]

**PsycINFO (APA)**

Search conducted via APA PsycINFO. Date range: 2000–2024.

(“criminal offenders” OR “offender psychotherapy” OR “forensic psychotherapy” OR “psychological treatment of offenders” OR “inmate*” OR “prisoner*” OR “antisocial personality disorder” OR “sex offender*” OR “violent offender*”) AND (“psychotherapy” OR “cognitive behavioral therapy” OR “cognitive behaviour therapy” OR “CBT” OR “schema therapy” OR “mentalisation-based treatment” OR “mentalization-based treatment” OR “MBT” OR “dialectical behavior therapy” OR “DBT” OR “therapeutic alliance” OR “clinical change processes”) AND (“treatment outcome” OR “change process*” OR “recidivism” OR “desistance” OR “rehabilitation” OR “recovery”)

**Web of Science Core Collection**

Search conducted via Web of Science Core Collection. Date range: 2000–2024. Topic search (TS) applied.

TS=(“criminal offenders” OR “offender psychotherapy” OR “forensic psychotherapy” OR “psychological treatment of offenders” OR “inmate*” OR “prisoner*” OR “antisocial personality disorder” OR “sex offender*” OR “violent offender*”) AND TS=(“psychotherapy” OR “cognitive behavioral therapy” OR “cognitive behaviour therapy” OR “CBT” OR “schema therapy” OR “mentalisation-based treatment” OR “mentalization-based treatment” OR “MBT” OR “dialectical behavior therapy” OR “DBT” OR “therapeutic alliance” OR “clinical change processes”) AND TS=(“treatment outcome” OR “change process*” OR “recidivism” OR “desistance” OR “rehabilitation” OR “recovery”)

**Scopus**

Search conducted via Scopus. Date range: 2000–2024. Title, abstract, and keyword search (TITLE-ABS-KEY) applied.

TITLE-ABS-KEY(“criminal offenders” OR “offender psychotherapy” OR “forensic psychotherapy” OR “psychological treatment of offenders” OR “inmate*” OR “prisoner*” OR “antisocial personality disorder” OR “sex offender*” OR “violent offender*”) AND TITLE-ABS-KEY(“psychotherapy” OR “cognitive behavioral therapy” OR “cognitive behaviour therapy” OR “CBT” OR “schema therapy” OR “mentalisation-based treatment” OR “mentalization-based treatment” OR “MBT” OR “dialectical behavior therapy” OR “DBT” OR “therapeutic alliance” OR “clinical change processes”) AND TITLE-ABS-KEY(“treatment outcome” OR “change process*” OR “recidivism” OR “desistance” OR “rehabilitation” OR “recovery”)

**PubMed/MEDLINE**

Search conducted via PubMed/MEDLINE. Date range: 2000–2024. MeSH terms and keywords combined.

(“criminal offenders”[Mesh] OR “offender psychotherapy” OR “forensic psychotherapy” OR “psychological treatment of offenders” OR “inmate*” OR “prisoner*” OR “antisocial personality disorder”[Mesh] OR “sex offender*” OR “violent offender*”) AND (“psychotherapy”[Mesh] OR “cognitive behavioral therapy”[Mesh] OR “cognitive behaviour therapy” OR “CBT” OR “schema therapy” OR “mentalisation-based treatment” OR “mentalization-based treatment” OR “MBT” OR “dialectical behavior therapy”[Mesh] OR “DBT” OR “therapeutic alliance” OR “clinical change processes”) AND (“treatment outcome”[Mesh] OR “change process*” OR “recidivism”[Mesh] OR “desistance” OR “rehabilitation”[Mesh] OR “recovery”)

**Criminal Justice Abstracts**

Search conducted via Criminal Justice Abstracts (EBSCO). Date range: 2000–2024.

(“criminal offenders” OR “offender psychotherapy” OR “forensic psychotherapy” OR “psychological treatment of offenders” OR “inmate*” OR “prisoner*” OR “antisocial personality disorder” OR “sex offender*” OR “violent offender*”) AND (“psychotherapy” OR “cognitive behavioral therapy” OR “cognitive behaviour therapy” OR “CBT” OR “schema therapy” OR “mentalisation-based treatment” OR “mentalization-based treatment” OR “MBT” OR “dialectical behavior therapy” OR “DBT” OR “therapeutic alliance” OR “clinical change processes”) AND (“treatment outcome” OR “change process*” OR “recidivism” OR “desistance” OR “rehabilitation” OR “recovery”)
