# Supplementary material for: Change process in psychotherapy for criminal offenders: a comprehensive review with content analysis
Source: Front Psychol. 2026 May 29;17:1776909. doi: 10.3389/fpsyg.2026.1776909 (PMC13260342; doi:10.3389/fpsyg.2026.1776909)
Supplement: Supplementary file 3 [file Supplementary_file_3.DOCX]

**Supplementary Material C: Coding Manual**

**C.1. Introduction**

This coding manual was developed for the content analysis reported in “Change Process in Psychotherapy for Criminal Offenders: A Comprehensive Review with Thematic Analysis.” The coding framework comprises 7 core themes and 32 subcomponents, derived inductively from the literature and guided by established theoretical concepts (e.g., Risk-Need-Responsivity model, Cognitive Behavioral Therapy, Good Lives Model). This manual provides operational definitions, keyword lists, coding rules, and examples of borderline cases to ensure transparency and replicability.

**C.2. Coding Rules**

The following rules were applied during the coding process:

1. **Full-Text Reading:** Coders read the full text of each included study. Keyword searches in Excel were used as an initial screening tool; however, each potential hit was verified by reading the surrounding text to confirm contextual relevance.
2. **Context Verification:** A keyword was counted as a “hit” only if it appeared in the context of discussing therapeutic change processes. For example, the term “warmth” was coded only if it referred to therapist characteristics or the therapeutic relationship, not if it described physical temperature or environmental conditions.
3. **Synonym Strategy:** In addition to the listed keywords, coders were trained to identify conceptually equivalent terms. For example, for “Empathy, Warmth, and Respect,” terms such as “supportive,” “non-judgmental attitude,” and “therapist caring” were also considered if contextually appropriate.
4. **Multiple Coding:** A single study could contribute to multiple subcomponents if it addressed more than one therapeutic component. No hierarchical weighting was applied.
5. **False Positives and Negatives:** False positives (keywords appearing in reference lists or contexts unrelated to therapeutic change) were excluded. False negatives were minimized through coder training on conceptual equivalence and the synonym strategy. Coding ambiguities were resolved through discussion between the two coders.
6. **Inter-Coder Reliability:** A randomly selected subset of 30 studies (18.4% of the total sample) was independently coded by two researchers. Inter-coder reliability, measured by Cohen's Kappa, was 0.92, indicating excellent agreement.

**C.3. Subcomponent Definitions and Keyword Lists**

**Theme I: Therapeutic Relationship and Alliance**

| Subcomponent | Definition | Keywords |
| --- | --- | --- |
| **I.1. Empathy, Warmth, and Respect (EWR)** | Therapist behaviors and attitudes reflecting genuine care, understanding, acceptance, and non-judgmental regard toward the client. This includes emotional attunement, validation, and treating the client as a person of worth regardless of their offense history. | empathy, warm*, unconditional positive regard, respect*, non-judgmental, acceptance, genuine, caring, compassion*, validation, attunement |
| **I.2. Trust and Safety Feeling** | The client's perception of the therapeutic environment as safe, reliable, and trustworthy. This includes feeling secure enough to disclose personal information and engage in difficult emotional work. | trust, safety, secure base, containment, reliable, predictable, holding environment |
| **I.3. Collaboration and Setting Shared Goals** | The process of establishing a collaborative working relationship where therapist and client agree on treatment goals and tasks. This includes shared decision-making and mutual engagement in the therapeutic process. | collaboration, collaborative, shared goals, goal setting, mutual, cooperative, working alliance, agreement |
| **I.4. Structured and Directive Therapy (SDT)** | The use of structured, manualized, and directive treatment approaches. This includes clear session frameworks, adherence to treatment protocols, and therapist-directed interventions. | structured, manualized, directive, program integrity, fidelity, protocol, framework, session structure |
| **I.5. Supportive Challenging Rather than Confrontation** | A therapeutic stance that involves firm but supportive challenges to client behaviors and beliefs, avoiding harsh or aggressive confrontation. This balances accountability with empathy. | supportive challenging, firm but fair, nonconfrontational, gentle challenge, constructive confrontation, supportive |

**Theme II: Motivation and Readiness to Change**

| Subcomponent | Definition | Keywords |
| --- | --- | --- |
| **II.1. Interventions Tailored to Stages of Change** | Interventions that match the client's readiness to change, as conceptualized by the Transtheoretical Model (precontemplation, contemplation, preparation, action, maintenance). | stages of change, transtheoretical, readiness, precontemplation, contemplation, action, maintenance |
| **II.2. Motivational Interviewing Techniques** | A client-centered, directive method for enhancing intrinsic motivation to change by exploring and resolving ambivalence. | motivational interviewing, MI, change talk, ambivalence, motivation, enhancing motivation |
| **II.3. Building Hope and Optimism** | Interventions aimed at fostering hope, future orientation, and belief in the possibility of positive change. | hope, optimism, future orientation, positive expectations, agency, pathways thinking |
| **II.4. Setting Positive Goals with the Good Lives Model (GLM)** | Using the Good Lives Model framework to help clients identify and pursue primary human goods (e.g., relatedness, mastery, autonomy) as prosocial goals. | Good Lives Model, GLM, primary goods, strengths-based, positive goals, human needs, well-being |
| **II.5. Combining External Incentives with Internal Motivation** | The use of external contingencies (e.g., court mandates, parole requirements) alongside efforts to cultivate internal motivation for change. | court-mandated, external motivation, extrinsic, coercion, incentives, contingency, mandated treatment |

**Theme III: Cognitive and Emotional Regulation Components**

| Subcomponent | Definition | Keywords |
| --- | --- | --- |
| **III.1. Identifying and Modifying Cognitive Distortions (IMCD)** | Interventions targeting offense-supportive beliefs, rationalizations, and justifications that sustain criminal behavior. This includes cognitive restructuring. | cognitive distortion*, offense-supportive attitudes, rationalization*, justification*, cognitive restructuring, thinking errors |
| **III.2. Processing Early Maladaptive Schemas** | Working with deep-seated, enduring patterns of thought and behavior (schemas) originating from early life experiences, often targeted in Schema Therapy. | early maladaptive schema*, EMS, schema therapy, schema mode*, schema processing |
| **III.3. Developing Emotion Regulation Skills (DERS)** | Interventions aimed at improving the capacity to identify, tolerate, and modulate emotional states, reducing impulsivity and affective dyscontrol. | emotion regulation, affect regulation, emotional control, impulse control, managing emotions |
| **III.4. Mindfulness and Acceptance Techniques** | Interventions using mindfulness, acceptance, and distress tolerance strategies to help clients observe thoughts and feelings without judgment or reactive behavior. | mindfulness, acceptance, distress tolerance, present-focused, non-judgmental awareness, DBT skills |
| **III.5. Enhancing Mentalization Capacity** | Interventions aimed at improving the ability to understand one's own and others' mental states (thoughts, feelings, intentions), often targeted in Mentalization-Based Treatment. | mentalization, mentalizing, MBT, theory of mind, reflective functioning, perspective-taking |

**Theme IV: Behavioural and Social Skill Components**

| Subcomponent | Definition | Keywords |
| --- | --- | --- |
| **IV.1. Social Skills Training (SST)** | Structured interventions teaching interpersonal skills, communication, assertiveness, and prosocial interaction. | social skills, interpersonal skills, communication training, assertiveness, prosocial behavior |
| **IV.2. Problem Solving and Decision Making Skills (PSDMS)** | Interventions teaching structured approaches to identifying problems, generating solutions, evaluating consequences, and making decisions. | problem-solving, decision making, cognitive skills, social problem-solving, reasoning |
| **IV.3. Anger Management and Aggression Control** | Interventions specifically targeting anger arousal, aggression, and violent behavior through cognitive-behavioral techniques. | anger management, aggression control, anger, violence prevention, impulse control |
| **IV.4. Developing Relapse Prevention Plans (DRPP)** | Interventions focused on identifying high-risk situations and developing concrete strategies to prevent reoffending, based on the Relapse Prevention model. | relapse prevention, RP, high-risk situations, coping strategies, prevention plan, offense cycle |
| **IV.5. Social Integration Skills** | Interventions aimed at facilitating successful reintegration into the community, including vocational training, employment support, and building prosocial networks. | reintegration, community integration, vocational training, employment, housing, prosocial network |

**Theme V: Group and Environmental Support Components**

| Subcomponent | Definition | Keywords |
| --- | --- | --- |
| **V.1. Group Cohesion and Solidarity** | The sense of belonging, mutual support, and shared purpose among group members in therapeutic settings. | group cohesion, group solidarity, therapeutic climate, peer support, group process |
| **V.2. Community-Based Support Networks** | Interventions that engage community resources, volunteers, or natural support systems to assist with reintegration and desistance. | community support, Circles of Support and Accountability, COSA, volunteers, community-based |
| **V.3. Family and Social Environment Involvement** | Engaging family members or significant others in treatment to address family dynamics and strengthen social support. | family involvement, family therapy, multisystemic, social ecology, family support |
| **V.4. Institutional Climate and Safety Feeling** | The perceived safety and therapeutic quality of the institutional environment (prison, hospital), including staff-client relationships and physical safety. | institutional climate, safety, containment, prison climate, ward atmosphere, therapeutic milieu |

**Theme VI: Therapist Characteristics and Quality of Psychotherapy Implementation**

| Subcomponent | Definition | Keywords |
| --- | --- | --- |
| **VI.1. Therapist Competence and Training (TCT)** | The skill, expertise, and formal training of therapists delivering interventions. | therapist competence, therapist training, expertise, qualification, skill |
| **VI.2. Therapist's Personal Values and Attitudes** | The personal values, beliefs, and attitudes that therapists bring to their work, including respect, non-judgmental stance, and commitment. | therapist values, attitudes, humanistic, belief, personal qualities, therapist characteristics |
| **VI.3. Therapeutic Fidelity and Manual Adherence** | The degree to which treatment is delivered as intended, adhering to manual specifications and program protocols. | fidelity, manual adherence, program integrity, treatment integrity, adherence |
| **VI.4. Supervision and Support Systems** | Clinical supervision, peer support, and organizational structures that support therapist well-being and professional development. | supervision, clinical supervision, support systems, peer support, burnout prevention |

**Theme VII: Individualised Adaptation (Responsivity)**

| Subcomponent | Definition | Keywords |
| --- | --- | --- |
| **VII.1. Intensity Adjustment Based on Risk Level (IABRL)** | Matching treatment intensity to the client's risk level, in accordance with the Risk principle (higher risk = higher intensity). | risk principle, intensity, dosage, risk level, treatment intensity, matching |
| **VII.2. Sensitivity to Cultural, Gender-Specific Needs** | Adapting interventions to account for cultural background, gender, ethnicity, and other identity factors that influence treatment engagement and effectiveness. | cultural sensitivity, cultural competence, gender-responsive, cultural adaptation, ethnicity |
| **VII.3. Adaptations for Psychopathy and Personality Disorders** | Modifying treatment approaches to address the specific needs and responsivity challenges of individuals with psychopathy or severe personality disorders. | psychopathy adaptation, ASPD, personality disorder, forensic adaptation, responsivity |
| **VII.4. Working with Trauma History** | Interventions specifically designed to address trauma exposure, post-traumatic stress symptoms, and their relationship to offending behavior. | trauma-focused, trauma history, PTSD, trauma processing, adverse childhood experiences |

**C.4. Examples of Borderline Cases and Resolution**

Below are examples of boundary cases encountered during coding and how they were resolved:

| Case | Description | Decision |
| --- | --- | --- |
| **Case 1** | “The therapist maintained a warm room temperature.” | **Not coded** under I.1 (Empathy, Warmth, and Respect) because the term “warm” referred to physical environment, not therapist interpersonal style. |
| **Case 2** | “The program included social skills training sessions focused on job interviews.” | **Coded** under IV.1 (Social Skills Training) only if the context indicated it was part of a therapeutic intervention for offenders; if it was purely vocational training without therapeutic content, it was **excluded**. |
| **Case 3** | “The therapeutic community emphasized trust among members.” | **Coded** under V.1 (Group Cohesion and Solidarity) if the context referred to group dynamics; **coded** under I.2 (Trust and Safety Feeling) if it referred to the individual's perception of safety. When both applied, the study was **coded for both** subcomponents. |
| **Case 4** | “The treatment was structured and followed a manual.” | **Coded** under I.4 (Structured and Directive Therapy) regardless of whether the manual was explicitly named. |
| **Case 5** | “The therapist was described as 'warm and caring' in the context of building rapport.” | **Coded** under I.1 (Empathy, Warmth, and Respect). The synonym "caring" was accepted as conceptually equivalent to "warmth." |
| **Case 6** | “The study mentioned 'relapse prevention' only in the introduction but did not discuss it as a treatment component.” | **Not coded** under IV.4 (Developing Relapse Prevention Plans) because the concept was not substantively addressed in the context of therapeutic change. |

**C.5. Reliability Information**

- **Inter-coder reliability:** A randomly selected subset of 30 studies (18.4% of the total sample) was independently coded by two researchers.
- **Cohen's Kappa:** 0.92 (mean across all 32 subcomponents).
- **Range of κ values:** 0.79 (Building Hope and Optimism) to 0.97 (Structured and Directive Therapy).
- **Software:** Microsoft Excel with manual verification.
